# Enemies make you stronger: Coevolution between fruit fly host and bacterial pathogen increases postinfection survivorship in the host

**DOI:** 10.1002/ece3.7774

**Published:** 2021-06-22

**Authors:** Neetika Ahlawat, Manas Geeta Arun, Komal Maggu, Nagaraj Guru Prasad

**Affiliations:** ^1^ Department of Biological Sciences Indian Institute of Science Education and Research Mohali Mohali India

**Keywords:** *Drosophila melanogaster*, experimental evolution, host‐pathogen coevolution, *Pseudomonas entomophila*

## Abstract

Multiple laboratory studies have evolved hosts against a nonevolving pathogen to address questions about evolution of immune responses. However, an ecologically more relevant scenario is one where hosts and pathogens can coevolve. Such coevolution between the antagonists, depending on the mutual selection pressure and additive variance in the respective populations, can potentially lead to a different pattern of evolution in the hosts compared to a situation where the host evolves against a nonevolving pathogen. In the present study, we used *Drosophila melanogaster* as the host and *Pseudomonas entomophila* as the pathogen. We let the host populations either evolve against a nonevolving pathogen or coevolve with the same pathogen. We found that the coevolving hosts on average evolved higher survivorship against the coevolving pathogen and ancestral (nonevolving) pathogen relative to the hosts evolving against a nonevolving pathogen. The coevolving pathogens evolved greater ability to induce host mortality even in nonlocal (novel) hosts compared to infection by an ancestral (nonevolving) pathogen. Thus, our results clearly show that the evolved traits in the host and the pathogen under coevolution can be different from one‐sided adaptation. In addition, our results also show that the coevolving host–pathogen interactions can involve certain general mechanisms in the pathogen, leading to increased mortality induction in nonlocal or novel hosts.

## Graphical abstract

Evolution of immune responses in a host evolving against a non‐evolving pathogen versus a host coevolving with a pathogen might be different. Using experimental evolution, we show that a coevolving host (*Drosophila melanogaster*) evolved higher survivorship postinfection with a coevolving or a non‐evolving pathogen (*Pseudomonas entomophila*) compared to a host that evolved against a non‐evolving pathogen. The coevolving pathogen evolved the ability to induce higher mortality even in non‐local hosts compared to its ancestor.

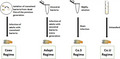

## 1 INTRODUCTION

Insects in the wild encounter a wide range of pathogens during their lifetime (Lazzaro et al., 2004). Generating an immune response to combat pathogenic attack is critical to an organism's fitness. Studies have reported the presence of considerable genetic variation in these immune responses in the wild and in laboratory‐adapted populations of insects (Lazzaro et al., 2004; Tinsley et al., 2006). There are several laboratory studies that have shown that these immune responses can evolve in populations of insects leading to better survival against the pathogen. Majority of these laboratory studies have evolved an insect host against a static or nonevolving pathogen. For instance, experimentally evolving populations of red flour beetle *Tribolium castaneum* against a bacterium *Bacillus thuringiensis* led to the evolution of divergent immune strategies in the host (Khan et al., 2017). Evolved responses in greater wax moth *Galleria mellonella* against *B. thuringiensis* included epigenetic mechanisms that helped the host to adapt against the pathogen (Mukherjee et al., 2017). Leitão et al. (2020) selected *Drosophila melanogaster* populations for increased survivorship against infection by a parasitoid wasp *Leptopilina boulardi*. The selected populations evolved upregulation of immune‐inducible genes and differentiation of lamellocyte precursors even under uninfected condition. Another study showed that *D. melanogaster* populations rapidly evolved improved resistance against an endoparasite *Asobara tabida* at the cost of reduced larval competitive ability (Kraaijeveld & Godfray, 1997). On the other hand, other laboratory studies showed no cost of evolved immune responses in terms of other life‐history traits in *D. melanogaster* populations evolved against a bacterial pathogen (Faria et al., 2015; Gupta et al., 2016). These studies described above are vital for elucidating the mechanisms underlying the evolution of host immunocompetence and associated life‐history costs. It is important to note that all these studies have focused on one‐sided evolution of host immunocompetence against a *static* or *nonevolving* pathogen (Faria et al., 2015; Gupta et al., 2016; Khan et al., 2017; Kraaijeveld & Godfray, 1997; Leitão et al., 2020; Mukherjee et al., 2017). However, host adaptation to a nonevolving pathogen may not represent an ecologically realistic scenario, since pathogens are expected to evolve rapidly to evade host defense mechanisms.

A more ecologically relevant scenario could be represented by the studies where the host and the pathogen coevolve with each other. In such a scenario where the interacting host and the pathogen coevolve, the selection imposed by the pathogen on the host might be entirely different from a scenario involving one‐sided evolution in the host against a static pathogen. Therefore, when compared with one‐sided host evolution, host–pathogen coevolution may lead to different outcomes. For example, as a consequence of change in virulence of the coevolving pathogen, it is possible that the coevolving host experiences stronger or variable selection compared to a host evolving against a static or nonevolving pathogen (Agrawal & Lively, 2002; Duxbury et al., 2019; Woolhouse et al., 2002; Zaman et al., 2014). If the coevolving pathogen evolves to become more virulent, it would exert stronger selection on the host. Therefore, the coevolving host's resistance against the pathogen may evolve at a faster rate relative to the host adapted to a nonevolving or static pathogen. On the other hand, if the pathogen's virulence oscillates across time, it is not guaranteed that the coevolving host would become more resistant against the pathogen. Additionally, coevolutionary interactions could lead to responses that are specific to their sympatric antagonists (Lively & Dybdahl, 2000; Morran et al., 2014). It must be noted, however, that empirical support for specific responses between the coevolving host and the pathogen is mixed with some studies finding evidences for specific responses (Brockhurst et al., 2014; Koskella et al., 2011; Morran et al., 2014) while others find no such specificity (Bérénos et al., 2012; Castledine et al., 2020; Morran et al., 2014). Nevertheless, it is clear that the outcomes of host–pathogen coevolutionary processes can be different from those of host adaptation to a static pathogen in many ways.

There are a few studies that have investigated the consequences of host–pathogen coevolutionary interactions. For instance, coevolutionary interactions between a water flea *Daphnia magna* and a bacterial endoparasite *Pasteuria ramosa* from lake sediments showed that over a period of time, the coevolving parasite decreased its virulence (Decaestecker et al., 2007). A laboratory coevolutionary study between red flour beetle *T. castaneum* host and a microparasite *Nosema whitei* showed increased host resistance and decreased pathogen virulence after several generations of coevolution (Bérénos et al., 2009). Another coevolutionary study using *T. castaneum* host and a fungal pathogen *Beauveria bassiana* reported increased pathogenic virulence against the immune secretions of coevolving host (Rafaluk et al., 2017). However, the host's survivorship was similar across pathogenic infection with ancestral and evolved fungal populations (Rafaluk‐Mohr et al., 2018). While these studies have measured specific traits of the coevolving host and pathogen, a few other studies have used host–pathogen interactions to address broader questions of pathogen infectivity and genetic variation in susceptibility to pathogens. *Wolbachia* is known to have inhibitory effects on the growth of RNA viruses within the hosts. Martinez et al. (2019) investigated the evolution of Drosophila C virus in *D. melanogaster* hosts infected with *Wolbachia* and found that DCV fail to evolve counter‐adaptations against *Wolbachia's* inhibitory effect. A study of four different species of *Drosophila* and their native viral isolates indicated that fly populations show greater genetic variation in susceptibility to viruses that they have coevolved with (Duxbury et al., 2019). We should note that none of these studies had an experimental treatment where host adapted against a nonevolving pathogen. Therefore, it becomes important to study both these evolutionary processes in a common experimental set up by directly comparing host evolution against a static pathogen versus host evolution against an evolving pathogen. Comparing both these processes using a common experimental set‐up would provide a clear picture of the possible differences in the outcomes of these two processes (one‐sided host adaptation and host–pathogen coevolution). Few of the studies investigating both these processes in a common experimental set‐up, primarily focused on *C. elegans* as the host, have reported that populations adapted to a static pathogen evolved a different suite of characters compared to populations that coevolved with their pathogen (Masri et al., 2015; Morran et al., 2011).

In the present study, using a common experimental set‐up we investigated (a) coevolution between the host *D. melanogaster* and the pathogen *Pseudomonas entomophila* and (b) one‐sided evolution of *D. melanogaster* host against a nonevolving *P. entomophila* pathogen. We set up four experimental evolution regimes:

1. Coevolution (Coev) (Both host and pathogen coevolve)
2. Adaptation (Adapt) (only host evolves in response to a nonevolving pathogen)
3. Sham control (Co.S)
4. Unhandled control (Co.U)

Each of these regimes had four independent replicate populations. Thus, the study consisted of 16 independent populations. After 20 generations of coevolution between the host and the pathogen, we asked the following questions‐

1. Are there any evolved changes in the Coev (coevolving host) and Adapt (one‐sided host adaptation) regimes in terms of survival against the coevolving pathogen and a static pathogen? Are these evolved changes different between Coev and Adapt hosts?
2. Are there any evolved changes in the coevolving pathogens in terms of inducing mortality in the host relative to the ancestral pathogen? If so, are the evolved changes specific to their local host?

## 2 MATERIALS AND METHODS

### 2.1 Model system

Our study involved four selection regimes. All these regimes were derived from large (*N* > 2,500 breeding adults per generation), laboratory‐adapted ancestral populations of *Drosophila melanogaster* called BRB populations (Blue Ridge Baseline populations), described in detail by Gupta et al. (2016). There are five independent replicate populations of BRB (labeled BRB 1‐5). We used four of those populations (BRB 1‐4) to derive the selection regimes. All the BRB populations are maintained on a 14‐day discrete generation cycle at 25°C temperature, 50%–60% RH, 12:12 LD cycle, on standard banana–yeast‐jaggery medium.

### 2.2 Ancestral pathogen

We used *Pseudomonas entomophila* (Pe), a species of gram‐negative, rod‐shaped bacteria originally isolated from *D. melanogaster* (Dieppois et al., 2015) as the pathogen. The bacterial strain (provided to us by Prof. Bruno Lemaitre) is maintained at −80°C, and the strain carries ampicillin and rifampicin resistance genes, along with a GFP tagged plasmid. Our initial experiments (prior to starting the selection regimes) showed that this pathogen is virulent to the flies (when infected at a bacterial optical density (OD_600_) of 0.5) and causes around 60% mortality. This single stock of *P. entomophila* is referred to as ‘ancestral Pe or Anc Pe’. This stock is the ancestor for all the coevolving pathogens used in the coevolution selection regime (see below). This stock also provides the nonevolving or static pathogen used to infect the Adaptation selection regime every generation (see below).

### 2.3 Selection regimes

**FIGURE 1:**
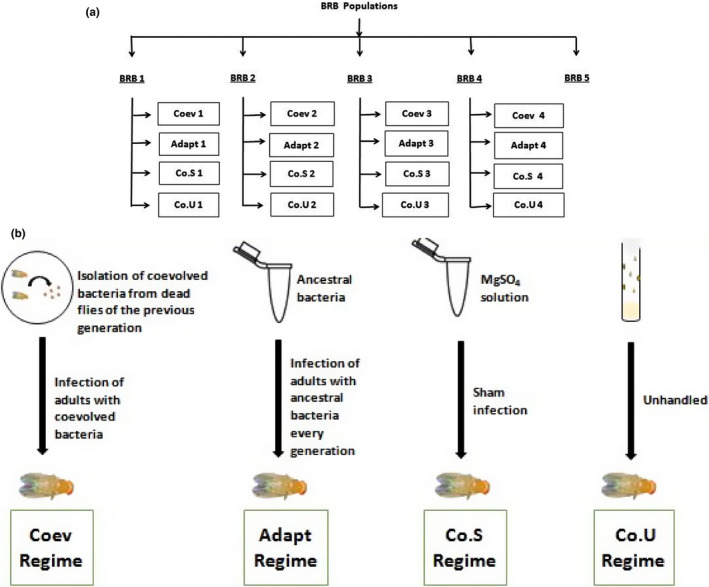
Schematic representation of the selection regimes. (a) the derivation of the selection regimes from ancestral BRB (Blue Ridge Baseline) populations, (b) the maintenance of each selection regime

Figure 1 shows a schematic of the derivation and maintenance of the selection regimes. From each of the four BRB populations (BRB 1‐4), we derived four selection regimes‐

1. Coevolution (Coev 1–4): both host and pathogen coevolve,
2. Adaptation (Adapt 1–4): only host evolves in response to a nonevolving pathogen,
3. Sham control (Co. S 1–4): injury control,
4. Unhandled control (Co.U 1–4): untreated control

In each of the selection regime, there were four replicate populations labeled 1–4 (discussed below) and these populations were maintained under conditions similar to those of BRB populations, except that these populations were cultured on a 16‐day discrete generation cycle. From each population, eggs were collected in 10 vials, each containing 6–7 ml of food, at a density of 70 eggs per vial. On the 12^th^ day post egg collection, (by which time around 95% of the flies had enclosed from pupae and were roughly 2–3 days old as adults) the flies in each selection regime were subjected to their selection regime‐specific treatment as described below. Post‐treatment, the flies from each selection regime were transferred to a Plexiglas cage (14 cm length*16 cm width*13 cm height) containing a food plate and were incubated at standard conditions. Fresh food plates were provided to the cages on alternate days. Later, on 16^th^ day post egg collection, a fresh food plate was provided to each cage to obtain eggs for next generation from the surviving flies. 18 hr later, we collected eggs from this plate at a density of 70 eggs per vial and 10 such vials were set up for each population to start the next generation.

#### 2.3.1 Coev (Coevolution regime)

On 12^th^ day post egg collection, we anesthetized the flies in each vial and randomly chose 20 males and 20 females from each vial to be infected with the coevolving pathogen. Thus, a total of 200 males and 200 females were infected per population per generation. We infected the flies by pricking them on the thorax (Khalil et al., 2015) with a minutein needle (0.01 mm, Fine Science Tools, CA) dipped in a bacterial suspension of coevolving pathogen *P. entomophila* (ampR and GFP tagged) at an optical density (OD_600_) of 0.40. The infected flies were then transferred to the cage. The infected flies began to die about 12 hr after infection with peak mortality happening between 24 and 48 hr. During the peak mortality period, dead flies were counted and 10–15 of those from each sex were collected and stored at 4℃. These flies were used to isolate bacteria to be used to infect the next generation. Mortality of the flies was monitored 2–3 times per day for four days (96 hr) postinfection. After this time, we did not observe any significant change in survivorship of the flies (Gupta et al., 2013). Four‐day postinfection (i.e., the 16th day post egg collection), there were roughly 200 flies alive in the cage and these survivors contributed to the next generation.

##### Preparation and isolation of coevolved pathogen

As stated earlier, dead flies of both the sexes were isolated from cages during peak mortality period (24–48 hr after infection) and stored at 4°C. These flies were used to extract the pathogen to infect the hosts in the next generation. Out of the collected dead flies, five flies of each sex were randomly picked and washed in absolute ethanol to remove surface contamination. Afterwards, these flies were kept on a sterilized plate for a few minutes to let the ethanol evaporate. These flies were then transferred to a micro‐centrifuge tube along with 200 µl of sterile 10 mM MgSO_4_ solution (Gupta et al., 2013). The flies were crushed using a homogenizer and a pestle, and the extract was serially diluted 4 times (by factors of 10) in MgSO_4_ solution. After this, 100 µl of the diluted sample was plated on LB agar plates containing ampicillin. These plates were then incubated for 20–22 hr at 27°C. Later, the plates were checked for bacterial colonies and were stored at 4°C for infecting the next generation of hosts. To prepare the bacterial suspension for fly infections, we used a protocol similar to the one described below, except that the overnight primary culture was set up from the LB agar plates stored at 4°C.

Please note that the bacteria isolated from a given population of hosts were used to infect hosts of the same population in the next generation. For example, bacteria isolated from dead flies of Coev 1 would be used to infect hosts from Coev 1 in the next generation. Bacteria from Coev 1 were never used to infect any other Coev population (Coev 2, 3, or 4). This was the same for the bacteria and the hosts of all the other Coev populations (Coev 2, 3, and 4). It is important to note that the bacteria used to infect all the populations trace their ancestry back to ancestral Pe (see section on Ancestral pathogen).

Over the first five generations of coevolution, we found that infecting the hosts of the next generation with the bacteria isolated from the dead hosts of the previous generation led to increasingly high mortality. While we wanted to have 200 survivors to start each generation, we ended up between 180 and 200 survivors. Hence, starting generation five, we slightly altered the protocol in the following way‐ (a) we reduced the bacterial OD to 0.4, (b) bacteria isolated from the dead flies of generation 5 were used to infect adults of generation 6 and 7. Then, bacteria was isolated from dead adults of generation 7. This bacteria was then used to infect adults of generations 8 and 9 and so on. This ensured that we had 200 surviving adults to start the next generation.

#### 2.3.2 Adapt. (Adaptation regime)

On 12^th^ day post egg collection, we anesthetized the flies in each vial and randomly chose 15 males and 15 females from each vial to be infected with the nonevolving or ancestral Pe. Hence, a total number of 150 males and 150 females per population per generation were infected. We infected the flies in the same way as described above, at an OD of 0.5. Mortality of the flies was monitored for four days (96 hr) postinfection and flies that survived bacterial infection, contributed to the next generation. Typically, there were about 200 surviving flies at the time of egg collection for the next generation.

#### 2.3.3 Co. S (Sham control regime)

On 12^th^ day post egg collection, from each vial we randomly anesthetized 10 males and 10 females to be pricked with a needle dipped in sterile, 10 mM MgSO_4_ solution, maintaining a total number of 100 males and 100 females per population per generation. After that, these flies were then transferred to a cage and provided a food plate. Pricking flies with MgSO_4_ solution acts as a control and confers 0%–1% fly mortality (Gupta et al., 2016). Four‐days post‐treatment (i.e., the 16^th^ day post egg collection), eggs were collected for next generation from the surviving flies (typically close to 200 flies).

#### 2.3.4 Co. U (Unhandled or Untreated control regime)

On 12^th^ day post egg collection, from each vial we randomly anesthetized 10 males and 10 females and transferred these flies to a cage (containing a food plate) maintaining a total number of 100 males and 100 females per population per generation. Four‐days later, eggs were collected to start the next generation. There was no fly mortality in these populations, and the number of flies alive at the time of egg collection was close to 200.

Please note that the number of flies used for infection/sham treatments was different in the four regimes. Highest mortality postinfection occurred in the Coev populations, followed by the Adapt populations. There was no mortality in the Co.S and Co.U populations. We wanted to have 200 flies at the time of egg collection for each generation. Therefore, to account for the differential mortality postinfection/sham treatments the initial number of flies used in each regime was different.

### 2.4 Block design

Populations with common subscripts shared a common ancestry and were hence more closely related to each other compared to populations with different subscripts. For example, Coev 1, Adapt 1, Co.S 1, and Co.U 1 were all derived from BRB 1 and were hence more closely related to each other than any of them were to Coev 2, etc. Therefore, populations with common subscripts were treated as statistical blocks. The sixteen populations used in this study were grouped into four distinct blocks. For example, Coev 1, Adapt 1, Co.S 1, and Co.U 1 formed block 1. They were always handled together during selection and during experimentation.

### 2.5 Standardization of fly populations

To observe evolved responses in the host and the pathogen, survival assays were conducted after the 20^th^ coevolution cycle. Before starting the experiment, flies from each regime were standardized (Rose, 1984) to account for the nongenetic parental effects that might have affected the traits under study. Eggs were collected from all the stock populations under standard conditions. On the 12^th^ day post egg collection, flies were transferred into cages and provided with ad libitum food. The flies were not subjected to any selection pressure that generation. In other words, flies from all the selection regimes were maintained under similar conditions and were not given any pathogenic infection or sham treatment that generation. To generate flies for the experiments, each cage was supplied with a food plate smeared with live yeast paste for 48 hr to boost fecundity. After 48 hr, a fresh food plate was provided in each cage. After 18 hr, eggs were collected from these plates at a density of seventy eggs per vial, with forty vials being collected from each selection regime by block combination.

### 2.6 Bacterial infection

All the fly infections were performed following the protocol mentioned in Gupta et al. (2013). Using the glycerol stock of the bacteria stored at −80°C, we set up an overnight primary culture in a conical flask containing Luria Bertani (LB) medium. The next morning, we used this overnight culture to start a secondary culture. After allowing it to grow for 3–4 hr, we used the secondary culture to prepare the final suspension with the desired OD by dissolving the bacterial pellet in sterile 10 mM MgSO_4_ solution. This bacterial slurry was used to infect the flies.

In order to infect flies, they were pricked on the thorax under mild CO_2_ anesthesia using a fine minutein needle (0.01 mm Fine Science Tools, CA) dipped in the bacterial suspension or MgSO_4_ solution for sham infections (Khalil et al., 2015). Fly stocks from Coev and Adapt regimes were infected at an OD of 0.4 and 0.5, respectively, during regular maintenance, whereas for experiments all the fly infections were done at an OD 0.44.

### 2.7 Experiment 1: Host–pathogen coevolution experiment

After 20 coevolution cycles, we investigated the evolved changes in the Coev (coevolving host) and Adapt (one‐sided host adaptation) hosts in terms of survivorship against the coevolving pathogens and the ancestral Pe pathogen. The experiments were performed on separate days for each of the four blocks.

For each selection regime and block, we collected eggs from the standardized flies as described above. On the 12^th^ day post egg collection, flies (2–3 day old as adults) from each selection regime (within that particular block) were randomly divided into three experimental treatments:‐

1. Coevolved Pe treatment: Flies from all the selection regimes were infected with coevolving Pe of their block.
2. Ancestral Pe treatment: Flies from all the selection regimes were infected with ancestral Pe.
3. Control treatment: Flies were subjected to sham infection.

For each block × selection regime × treatment combination, 50 males and 50 females were chosen randomly. After infection (or sham infection), flies were immediately transferred to cages and were provided with fresh food plates (which were replaced with fresh ones two days later). We monitored host mortality postinfection in each cage, by recording deaths every 3–4 hr for the first 48 hr, and subsequently every 6–8 hr until 96‐hr postinfection. By this time, mortality due to bacterial infection ceased, and therefore, we stopped our observations (Gupta et al., 2013).

### 2.8 Experiment 2: Mortality of nonlocal hosts

To measure whether the change in coevolving pathogen's ability to induce host mortality was specific to their local hosts, we used two different laboratory‐adapted baseline populations called BRB‐5 and Canton‐S as novel hosts. These populations had never experienced bacterial infection previously. BRB‐5 population is genetically diverse and is related to BRB1‐4, having been derived from the same ancestral population as BRB 1–4, and had remained an independent population for about 160 generations. Canton‐S is an inbred population and was obtained from Dr. Sheeba Vasu's laboratory at JNCASR, Bangalore.

Canton‐S and BRB‐5 eggs were collected at a density of 70 per vial containing 6–7 ml food. On the 12^th^ day post egg collection, flies from each host population were divided into six infection treatments‐ (a) Ancestral Pe infection treatment, (b) infection with coevolving Pe from block 1, (c) infection with coevolving Pe from block 2, (d) infection with coevolving Pe from block 3, (e) infection with coevolving Pe from block 4, and (f) sham infection treatment using MgSO_4_. Mortality was monitored for 96‐hr postinfection.

The experiment was replicated twice and was conducted on different days. Therefore, we had 2 populations × 6 treatments × 100 individuals (50 male and 50 female flies) × 2 replicates; that is, a total of 2,400 flies were infected for the experiment.

### 2.9 Statistical analysis

All analyses were performed in R version 4.0.2. We analyzed the survivorship data from both experiments using Cox proportional hazards models implemented using the R package “coxme” (Therneau, 2020). For experiment 1, we fit the following model separately for males and females:

Time to death ~ Selection + Pathogen + Selection: Pathogen + (1|Block)

This model treats selection regime and pathogen as fixed factors, while blocks are treated as a random factor. In order to investigate variability across blocks, we also fitted the following model separately for each sex and each block:

Time to death ~ Selection + Pathogen + Section : Pathogen

For experiment 2, we fitted the following model separately for each host population (BRB 5 or Canton S) and each sex:

Time to death ~ Pathogen + (1|Replicate)

This model treats pathogen as a fixed factor, while independent replicates of the experiment are assumed to be random.

## 3 RESULTS

To test whether there was a primary response to selection, we measured survivorship of all the populations when infected with ancestral Pe after 10, 15, and 20 generations of selection. We found a significant effect of selection with males and females of both Adapt and Coev populations having better survivorship compared to the control populations. The detailed results are presented in the supplementary material (Figure S4, Table S7).

### 3.1 Experiment 1

**FIGURE 2:**
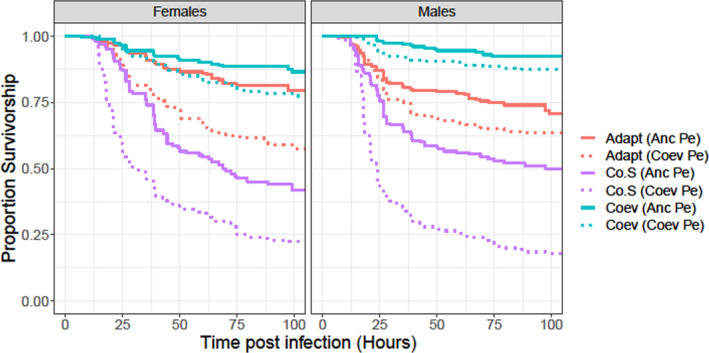
Survivorship curves for female and male hosts from Adapt (red), Co.S (purple), or Coev (blue) regimes infected with either ancestral pathogen (Anc Pe—solid curves) or the coevolving pathogens (Coev Pe—dotted curves) from the corresponding block after 20 cycles of coevolution (experiment 1)

After 20 cycles of coevolution, we infected male and female hosts from four different selection regimes (Adapt, Coev, Co.S, and Co.U) with either Anc Pe or the coevolving Pe (or Coev Pe) of their respective blocks and measured their survivorship postinfection. The complete analysis including all the selection regimes is presented in the supplementary material (Figure S2, Figure S3, Table S5). Excluding Co.U from our analysis does not change our results or conclusions. Since comparisons of Co.S with Adapt and Coev regimes (a) reveal evolved differences attributable to bacterial infection and (b) allow us to make a smaller number of comparisons, here we present analysis including Adapt, Coev and Co.S regimes only. Our results indicate that, irrespective of which pathogen they were infected with, both males and females from the Coev regime had the highest survivorship, males and females from the Adapt regime had intermediate survivorship, while males and females from the Co.S regime had the lowest survivorship postinfection (Figure 2). This pattern was also reflected in our Cox proportional hazards model. In both males and females, the hazard rate corresponding to the Co.S regime was significantly higher (which is equivalent to lower survivorship) than the hazard rate corresponding to the Adapt regime (which was constrained in the model to be 1; Table 1A,B). In males, the hazard rate corresponding to the Coev regime was significantly lower (which is equivalent to higher survivorship) than the hazard rate of the Adapt. regime (Table 1B). In females too, the hazard rate for the Coev regime was lower than the Adapt regime; however, this difference was not statistically significant. In both males and females, Coev Pe caused higher mortality in all three kinds of hosts relative to Anc Pe (Figure 2). In our Cox proportional hazards model, the hazard rate associated with Coev Pe was significantly higher (equivalent to lower survivorship of its hosts) than Anc Pe in both sexes (Table 1A,B). Additionally, the coefficient corresponding to the interaction term Selection Co.S: Pathogen Coev was also significant in males (Table 1B). This was a reflection of the fact that while Coev Pe induced higher mortality in all three kinds of male hosts relative to Anc Pe, it did so to a considerably higher degree in males from the Co.S regime (Figure 2).

**TABLE 1:** The output of Cox proportional hazards models for male and female hosts from Adapt, Coev, and Co.S regimes infected with either ancestral pathogen (Anc Pe) or the coevolving pathogens (Coev Pe) from the corresponding block (experiment 1). Hazard rates are expressed relative to the hazard rates of the default level of each fixed factor, which are constrained to be 1. The default level for “Selection” is Adapt, while the default level for “Pathogen” is Anc Pe. Lower CI and Upper CI indicate lower and upper bounds of 95% confidence intervals. Confidence intervals that do not contain 1 signify statistical significance and are shown in bold. Higher hazard rates are equivalent to lower survivorship in the hosts

Fixed coefficients	Hazard ratios	Lower CI	Upper CI
*(A) Females*			
Selection Co.S	3.8512	**2.6759**	**5.5429**
Selection Coev	0.6181	0.3741	1.0212
Pathogen Coev Pe	2.6582	**1.8147**	**3.8938**
Selection Co.S: Pathogen Coev Pe	0.8561	0.5447	1.3453
Selection Coev: Pathogen Coev Pe	0.7161	0.3847	1.3330
Random effects	Variance		
Block	0.1374		
*(B) Males*			
Selection Co.S	2.1242	**1.5272**	**2.9544**
Selection Coev	0.2387	**0.1349**	**0.4226**
Pathogen Coev Pe	1.4289	**1.0066**	**2.0284**
Selection Co.S: Pathogen Coev Pe	1.8262	**1.1863**	**2.8112**
Selection Coev: Pathogen Coev Pe	1.2257	0.5909	2.5424
Random effects	Variance		
Block	0.0308		

Our separate analyses for each block showed patterns that are largely consistent with the overall analysis with some exceptions. Typically, in both sexes the Coev hosts had the highest survivorship postinfection by pathogen of either kind, followed by the Adapt hosts; in both sexes, the Co.S hosts had the lowest survivorship postinfection (Figure S1). In block 3, however, Adapt females exhibited a marginally higher survivorship compared to Coev females when infected by Anc Pe (Figure S1b). Typically, Coev Pe induced higher mortality in all three kinds of hosts relative to Anc Pe; however, block 4 was a major exception to this trend (Figure S1). The block 4 Coev Pe did not cause higher mortality in its hosts compared to Anc Pe. The outputs of our Cox proportional hazards models fitted separately for each block and sex are summarized in Tables S1‐S4.

### 3.2 Experiment 2

**FIGURE 3:**
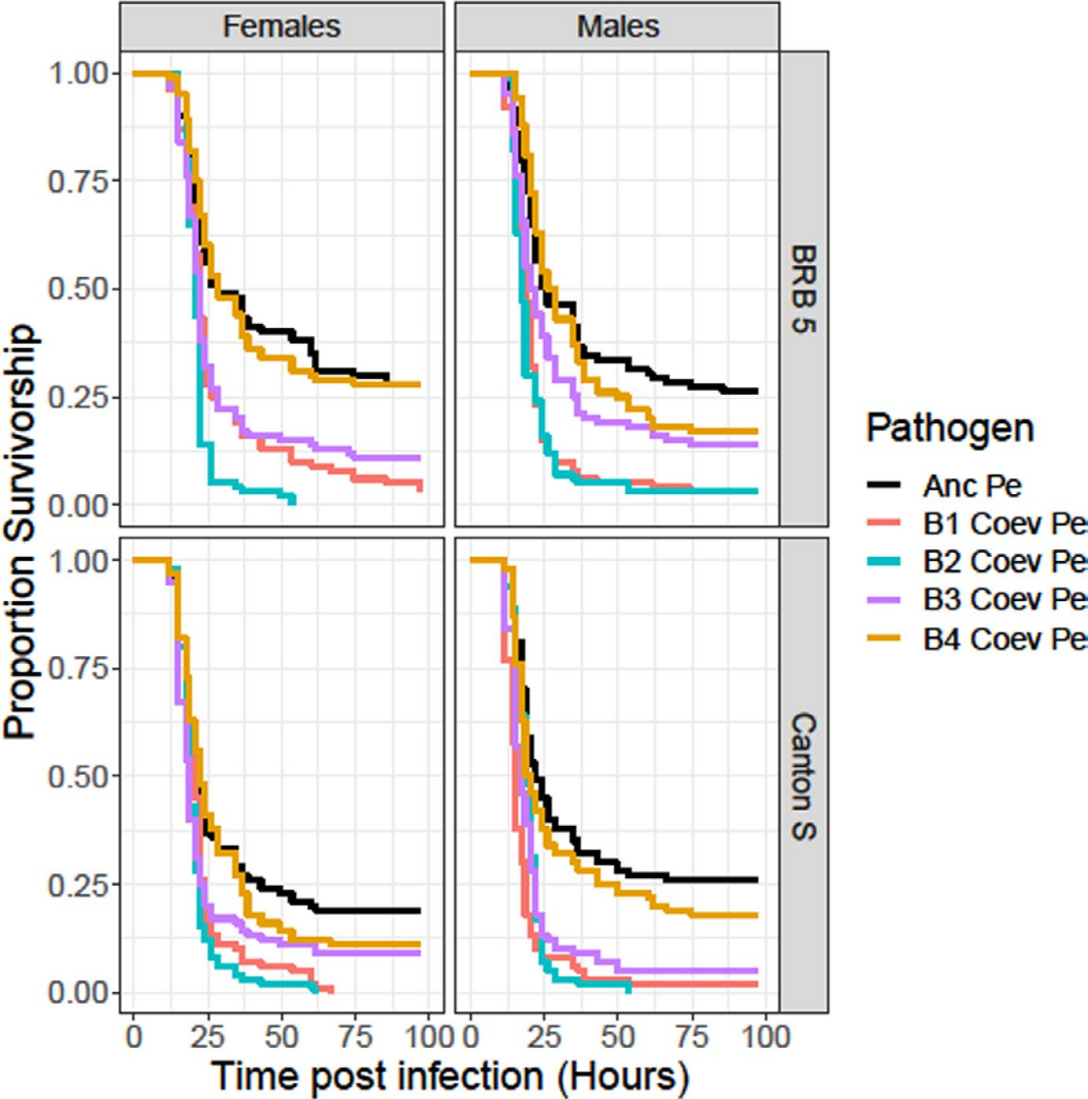
Survivorship curves for female and male hosts from the BRB 5 and Canton S populations infected with one of five different kinds of pathogens—ancestral pathogen (Anc Pe—black), block 1 coevolving pathogen (B1 Coev Pe—red), block 2 coevolving pathogen (B2 Coev Pe—blue), block 3 coevolving pathogen (B3 Coev Pe—purple) or block 4 coevolving pathogen (B4 Coev Pe—gold)—after 20 cycles of coevolution (experiment 2)

After 20 cycles of coevolution, we infected males and females from two nonlocal host populations (BRB 5 and Canton S) with each of the five different kinds of pathogens (Anc Pe, and Coev Pe pathogens from each of the four independent blocks) and measured their survivorship postinfection. Male and female hosts from both populations had reduced survivorship when infected with Coev Pe from block 1, block 2, and block 3, relative to when infected by Anc Pe (Figure 3). In our Cox proportional hazards models, for both populations, in males and in females, the hazard rates corresponding to Coev Pe from block 1, block 2, and block 3 were significantly higher (equivalent to lower survivorship in their hosts) than the hazard rate for Anc Pe (Tables 2 and 3). Consistent with our findings from experiment 1, Coev Pe from block 4 did not have a hazard rate that was significantly different from the hazard rate of Anc Pe (Figure 3; Tables 2 and 3) in males and females from both the populations. Interestingly, there appeared to be variability among Coev Pe pathogens from the four blocks in terms of their ability to cause mortality in their hosts. Coev Pe from block 2 caused the highest mortality among male and female hosts from both populations, while Coev Pe from block 4 was, as described above, the most benign among the four Coev Pe pathogens (Tables 2 and 3). Coev Pe from block 1 and block 3 caused intermediate levels of mortality in their hosts, as also indicated by the fact that their hazard rates were lower than the hazard rate for Coev Pe from block 2, but higher than the hazard rate for coevolving Pe from block 4. Coev Pe from block 1 had a higher hazard rate compared to coevolving Pe from block 3.

**TABLE 2:** The output of Cox proportional hazards models for male and female hosts from the BRB 5 population infected with either ancestral pathogen (Anc Pe), or one of the four coevolving pathogens (Coev Pe) (from each of the four independent blocks) (experiment 2). Hazard rates are expressed relative to the hazard rate of the default level, which is constrained to be 1. The default level for “Pathogen” is Anc Pe. Lower CI and Upper CI indicate lower and upper bounds of 95% confidence intervals. Confidence intervals that do not contain 1 signify statistical significance and are shown in bold. Higher hazard rates are equivalent to lower survivorship in the hosts

Fixed coefficients	Hazard ratios	Lower CI	Upper CI
*(A) BRB 5 Females*			
Pathogen B1 Coev Pe	2.1107	**1.5507**	**2.8729**
Pathogen B2 Coev Pe	3.3434	**2.4356**	**4.5896**
Pathogen B3 Coev Pe	1.9147	**1.4007**	**2.6174**
Pathogen B4 Coev Pe	0.9770	0.7046	1.3547
Random effects	Variance		
Replicate	<0.0001		
*(B) BRB 5 Males*			
Pathogen B1 Coev Pe	2.6502	**1.9451**	**3.6111**
Pathogen B2 Coev Pe	3.0531	**2.2398**	**4.1619**
Pathogen B3 Coev Pe	1.5508	**1.1347**	**2.1194**
Pathogen B4 Coev Pe	1.1122	0.8117	1.5238
Random effects	Variance		
Replicate	0.0001		

**TABLE 3:** The output of Cox proportional hazards models for male and female hosts from the Canton S population infected with either ancestral pathogen (Anc Pe), or one of the four coevolving pathogens (Coev Pe) (from each of the four independent blocks) (experiment 2). Hazard rates are expressed relative to the hazard rate of the default level, which is constrained to be 1. The default level for “Pathogen” is Anc Pe. Lower CI and Upper CI indicate lower and upper bounds of 95% confidence intervals. Confidence intervals that do not contain 1 signify statistical significance and are shown in bold. Higher hazard rates are equivalent to lower survivorship in the hosts

Fixed coefficients	Hazard ratios	Lower CI	Upper CI
*(A) Canton S Females*			
Pathogen B1 Coev Pe	1.7172	**1.2760**	**2.3110**
Pathogen B2 Coev Pe	2.2376	**1.6580**	**3.0198**
Pathogen B3 Coev Pe	1.6939	**1.2546**	**2.2870**
Pathogen B4 Coev Pe	1.1148	0.8246	1.5070
Random effects	Variance		
Replicate	0.0001		
*(B) Canton S Males*			
Pathogen B1 Coev Pe	3.5595	**2.6158**	**4.8436**
Pathogen B2 Coev Pe	2.2350	**1.6416**	**3.0430**
Pathogen B3 Coev Pe	2.2654	**1.6655**	**3.0812**
Pathogen B4 Coev Pe	1.2349	0.9018	1.6910
Random effects	Variance		
Replicate	<0.0001		

## 4 DISCUSSION

Using experimental evolution, we studied the coevolutionary process between an insect host *Drosophila melanogaster* and a bacterial pathogen *Pseudomonas entomophila* (Pe). Our results from survivorship assays conducted after 10, 15, and 20 generations of selection indicate that males and females from the Coev and Adapt populations evolved increased survivorship postinfection (Figure S4, Table S5). Across generations, flies from Coev populations tended to survive better than flies from Adapt populations. All these results clearly suggest that there was a strong primary response to selection.

After 20 generations of selection, we measured host's survivorship against pathogenic infection and pathogen's ability to induce mortality in the flies. Our study majorly found—(1) Compared to hosts adapting to a nonevolving pathogen (Adapt host), the Coev host on average, evolved higher survivorship postinfection with ancestral Pe or coevolving Pe; (2) after 20 coevolution generations, coevolving pathogens evolved increased ability to induce host mortality; (3) coevolving pathogens evolved to induce higher mortality in several nonlocal hosts (compared to the ancestral pathogen).

In our assays, we used a bacterial suspension with OD of 0.44 for infecting flies. Therefore, in the assays, flies from Adapt populations were exposed to slightly benign dose of pathogens while the Coev flies were exposed to slightly stronger dose of pathogens than what they are exposed to during regular maintenance (OD 0.5 and 0.4, respectively). However, this difference in infection dose is unlikely to explain our results since, flies from Adapt populations had lower survivorship in spite of a benign dose while flies from Coev populations had higher survivorship in spite of a higher dose. If anything, this could reduce the survivorship difference observed between the two selection regimes.

One plausible explanation for the increase in survivorship of coevolving hosts (Coev) compared to the hosts evolving against a static pathogen (Adapt) could be the stronger selection pressure imposed on the coevolving hosts by the coevolving pathogen. In our selection protocol for the coevolving populations, the bacteria were collected from the dead flies and used to infect flies from subsequent generations. Therefore, it is likely that in the coevolving populations, there was selection for bacteria that could induce greater mortality. Thus, flies in the coevolving populations would likely face challenge from more virulent bacteria every generation compared to flies in populations adapting to a static ancestral pathogen. Consistent with this idea, during selection, we observed greater mortality of flies in Coev (coevolving) populations compared to populations adapting to static ancestral pathogen. Thus, our coevolution populations probably represent a more ecologically relevant scenario where hosts are faced with more virulent coevolving pathogens every generation. However, a large number of laboratory studies have focused on one‐sided host evolution. There are a few studies that have compared one‐sided evolution and coevolution using the same experimental system. Working on the coevolutionary process between *C. elegans* and *B. thuringiensis*, Masri et al. (2015) reported that the coevolving pathogen became more virulent over time. Additionally, hosts that were coevolving with the pathogen had higher survival compared to the ancestral host when infected with the coevolving pathogen. Another study using *C. elegans* and *B. thuringiensis* found no difference in host killing ability in the bacterial populations from coevolution and evolution (against nonevolving host) treatment (Kloesener et al., 2017). In a study of coevolution between *Pseudomonas fluorescens* SBW25 bacteria and DNA phage phi2, the coevolving phage population had higher infectivity to a wider range of allopatric bacterial host colonies, as compared to the phage population evolving against nonevolving ancestral bacterial population (Gandon et al., 2008; Hall et al., 2011). Thus, our study, along with others (Buckling & Rainey, 2002; Hall et al., 2011; Kloesener et al., 2017; Masri et al., 2015), suggests that the ecologically meaningful coevolutionary scenario can lead to the evolution of a different pattern of traits compared to the patterns of traits that evolve under the more commonly used laboratory approach of one‐sided host evolution.

Results from our experiment 2 also indicate that the coevolving pathogens had probably evolved to induce higher mortality in their hosts. By coevolution generation 20, the coevolving pathogens had evolved the ability to induce higher mortality in nonlocal hosts from BRB‐5 and Canton‐S populations. While coevolving pathogens from different blocks varied in their ability to induce mortality in the hosts, the trend was clearly toward greater mortality induction by coevolving pathogens compared to ancestral pathogen. Our results also show that the coevolving pathogens had evolved higher mortality induction in a broad range of nonlocal host genotypes. Our results are broadly in agreement with a number of other studies. In a coevolutionary study between bacteria and its phage, the coevolving phage was observed to be virulent to allopatric hosts from other replicate populations and the virulence of the phages from each replicate population was also different (Buckling & Rainey, 2002). Poullain et al. (2008) observed an increased infectivity of the coevolving phage populations to their local and a wider range of nonlocal host genotypes relative to phage populations evolved against nonevolving bacterial population. Results from another study involving *Paramecium caudatum* and *Holospora undulate* show that the parasite infectivity was lesser for its sympatric host as compared to other allopatric or nonlocal hosts (Adiba et al., 2010). Thus, our results along with the results from multiple other studies seem to suggest that coevolving pathogens become better at infecting nonlocal hosts also.

Often coevolving pathogens are expected to evolve at a faster rate compared to their hosts, and therefore, evolution can happen at different rates in the host and the pathogen. However, the fitness of one antagonist depends on the strength of selection imposed by the other antagonist and the presence of adaptive genetic variation in the population (Gandon & Michalakis, 2002). In our study, we find that after 20 generations of coevolution, (a) the coevolving hosts have increased their ability to survive infection and (b) three out of the four coevolving pathogens have increased ability to induce mortality in local and nonlocal hosts. Therefore, given the design of our study, we cannot decipher whether the host or the pathogen is evolving at a faster rate.

While, the coevolving pathogens, overall, evolved to induce higher mortality in their hosts, one exception was the coevolving pathogen from block 4, which, even after 20 cycles of coevolution, was comparable to ancestral pathogen in terms of the mortality it induced. It is likely that this was a consequence of our experimental protocol for culturing the coevolving pathogens. In the maintenance protocol, the coevolving Pe pathogens were under fluctuating selection, as the coevolving bacteria experienced the following three phases of growth followed by bottlenecks every generation:

1. In the overnight LB culture grown to an OD_600_ of 2.0
2. In the secondary culture grown to an OD_600_ of 0.4
3. Inside the fly hosts that contribute to the next generation's pathogen

Within the first two days, while growing in the LB medium, there was, presumably, stronger selection for faster growth and continued ampicillin resistance. Recent theoretical and empirical work predicts that in asexual populations experiencing periodic bottlenecks, the extent of adaptation should depend upon the quantity *N*_0_/*g* (as opposed to the harmonic mean population size *N*_o_ × *g*) (where *N*_o_ is the bottleneck size, and *g* represents the number of generations of growth) (Chavhan et al., 2019). In this case, *g* is likely to be comparable (i.e., of the same order of magnitude) for each of the three phases, the estimates of *N*_0_ in LB (~10^7^) are likely to be several orders of magnitude higher than the estimates of *N*_0_ for growth in the 10 flies that contribute to the next generation's bacteria (~10^4^ when infections are performed at an OD_600_ of 0.4) (Gupta et al. (2013) and unpublished observations in our lab). Therefore, in our design, growth in LB is likely to make a significant contribution to the overall selection acting on the populations of coevolving pathogens. However, our results indicate that coevolving hosts evolved ability to induce higher mortality compared to ancestral pathogen even after the considerable influence of the growth phase in LB. Additionally, unlike the host populations in which selection was largely acting on standing genetic variation, selection on the coevolving pathogens (which trace their ancestry to an isogenic ancestral stock) was contingent on novel mutations (Kawecki et al., 2012). As a consequence, the virulence of the coevolving pathogens would increase only if there was a mutation that increased the virulence without significant costs to growth rate in LB. Given the ubiquity of performance trade‐offs across environments (Bataillon et al., 2011; Cooper & Lenski, 2000; Kassen, 2002; Remold, 2012), it is reasonable to assume that such mutations are rare. Alternatively, the virulence could increase whether a mutation that increased the virulence and imposed costs to growth in LB and/or ampicillin resistance was followed by a compensatory mutation that ameliorated those costs. Additionally, since every generation the cultures for the coevolving pathogens were set up using 10 colonies, there was an upper bound of 10 genotypes which selection could “see” in our set up. Therefore, rates of genetic drift in the populations of coevolving pathogens would have been appreciable. Evolution of improved virulence would then require not only genetic changes that appear to be rare, but also that these novel favorable genetic variants are not lost due to drift. We believe that there is a compelling case in favor of this model as it explains three key features of our results: (A) Evolution of improved virulence was slow. (B) There was stochasticity associated with evolution of virulence in replicate populations of coevolving pathogens. (C) When coevolving pathogens did evolve improved virulence (by coevolution generation 20), they did not incur any costs to their growth rates in LB (Ahlawat et al, manuscript under preparation).

Our results clearly show that the evolved traits of host and the pathogen in a coevolutionary process can be different from host evolution against a nonevolving pathogen. While coevolution is expected to be quite specific (Koskella et al., 2011; Morran et al., 2014) to a given set of host and pathogen, there are mixed results in this context (Bérénos et al., 2012; Castledine et al., 2020). Our results also suggest that such evolution can increase the virulence of the pathogens even in nonlocal or novel hosts, pointing to the involvement of generalized mechanisms in the evolution of higher host killing ability in the coevolving pathogens.

## CONFLICT OF INTEREST

The authors have no conflict of interest.

## AUTHOR CONTRIBUTION

**Neetika Ahlawat:** Conceptualization (equal); Formal analysis (equal); Investigation (lead); Writing‐original draft (lead); Writing‐review & editing (equal). **Manas Geeta Arun:** Formal analysis (lead); Writing‐review & editing (equal). **Komal Maggu:** Investigation (equal); Writing‐review & editing (equal). **N.G. Prasad:** Conceptualization (equal); Writing‐review & editing (equal).

### OPEN RESEARCH BADGES

This article has earned an Open Data Badge for making publicly available the digitally‐shareable data necessary to reproduce the reported results. The data is available at https://doi.org/10.5061/dryad.05qfttf2z.

## Supporting information

Supplementary Material

## Data Availability

Data from this manuscript are available: Dryad https://doi.org/10.5061/dryad.05qfttf2z

## References

[ece37774-bib-0001] Adiba, S. , Huet, M. , & Kaltz, O. (2010). Experimental evolution of local parasite maladaptation. Journal of Evolutionary Biology, 23(6), 1195–1205. 10.1111/j.1420-9101.2010.01985.x 20406349

[ece37774-bib-0002] Agrawal, A. , & Lively, C. (2002). Infection genetics: Gene‐for‐gene versus matching‐alleles models and all points in between. Evolutionary Ecology Research, 4, 79–90.

[ece37774-bib-0003] Bataillon, T. , Zhang, T. , & Kassen, R. (2011). Cost of adaptation and fitness effects of beneficial mutations in *Pseudomonas fluorescens* . Genetics, 189(3), 939–949. 10.1534/genetics.111.130468 21868607 PMC3213353

[ece37774-bib-0004] Bérénos, C. , Schmid‐Hempel, P. , & Wegner, K. M. (2009). Evolution of host resistance and trade‐offs between virulence and transmission potential in an obligately killing parasite. Journal of Evolutionary Biology, 22(10), 2049–2056. 10.1111/j.1420-9101.2009.01821.x 19732263

[ece37774-bib-0005] Bérénos, C. , Schmid‐Hempel, P. , & Wegner, K. M. (2012). Complex adaptive responses during antagonistic coevolution between *Tribolium castaneum* and its natural parasite *Nosema whitei* revealed by multiple fitness components. BMC Evolutionary Biology, 12, 11. 10.1186/1471-2148-12-11 22280468 PMC3305629

[ece37774-bib-0006] Brockhurst, M. A. , Chapman, T. , King, K. C. , Mank, J. E. , Paterson, S. , & Hurst, G. D. D. (2014). Running with the Red Queen: The role of biotic conflicts in evolution. Proceedings. Biological Sciences, 281(1797), 10.1098/rspb.2014.1382 PMC4240979 25355473

[ece37774-bib-0007] Buckling, A. , & Rainey, P. B. (2002). Antagonistic coevolution between a bacterium and a bacteriophage. Proceedings. Biological Sciences, 269(1494), 931–936. 10.1098/rspb.2001.1945 12028776 PMC1690980

[ece37774-bib-0008] Castledine, M. , Padfield, D. , & Buckling, A. (2020). Experimental (co)evolution in a multi‐species microbial community results in local maladaptation. Ecology Letters, 23(11), 1673–1681. 10.1111/ele.13599 32893477

[ece37774-bib-0009] Chavhan, Y. D. , Ali, S. I. , & Dey, S. (2019). Larger numbers can impede adaptation in asexual populations despite entailing greater genetic variation. Evolutionary Biology, 46(1), 1–13. 10.1007/s11692-018-9467-6

[ece37774-bib-0010] Cooper, V. S. , & Lenski, R. E. (2000). The population genetics of ecological specialization in evolving Escherichia coli populations. Nature, 407(6805), 736–739. 10.1038/35037572 11048718

[ece37774-bib-0011] Decaestecker, E. , Gaba, S. , Raeymaekers, J. A. M. , Stoks, R. , Van Kerckhoven, L. , Ebert, D. , & De Meester, L. (2007). Host‐parasite “Red Queen” dynamics archived in pond sediment. Nature, 450(7171), 870–873. 10.1038/nature06291 18004303

[ece37774-bib-0012] Dieppois, G. , Opota, O. , Lalucat, J. , & Lemaitre, B. (2015) Pseudomonas entomophila: A Versatile Bacterium with Entomopathogenic Properties. In J.‐L. Ramos , J. B. Goldberg , & A. Filloux (Eds.), Pseudomonas (pp. 25–49). Springer Netherlands. 10.1007/978-94-017-9555-5_2

[ece37774-bib-0013] Duxbury, E. M. , Day, J. P. , Maria Vespasiani, D. , Thüringer, Y. , Tolosana, I. , Smith, S. C. L. , Tagliaferri, L. , Kamacioglu, A. , Lindsley, I. , Love, L. , Unckless, R. L. , Jiggins, F. M. , & Longdon, B. (2019). Host‐pathogen coevolution increases genetic variation in susceptibility to infection. eLife, 8, 10.7554/eLife.46440 PMC6491035 31038124

[ece37774-bib-0014] Faria, V. G. , Martins, N. E. , Paulo, T. , Teixeira, L. , Sucena, É. , & Magalhães, S. (2015). Evolution of *Drosophila* resistance against different pathogens and infection routes entails no detectable maintenance costs. Evolution; International Journal of Organic Evolution, 69(11), 2799–2809. 10.1111/evo.12782 26496003

[ece37774-bib-0015] Gandon, S. , Buckling, A. , Decaestecker, E. , & Day, T. (2008). Host‐parasite coevolution and patterns of adaptation across time and space. Journal of Evolutionary Biology, 21(6), 1861–1866. 10.1111/j.1420-9101.2008.01598.x 18717749

[ece37774-bib-0016] Gandon, S. , & Michalakis, Y. (2002). Local adaptation, evolutionary potential and host–parasite coevolution: Interactions between migration, mutation, population size and generation time. Journal of Evolutionary Biology, 15(3), 451–462.

[ece37774-bib-0017] Gupta, V. , Ali, Z. S. , & Prasad, N. G. (2013). Sexual activity increases resistance against *Pseudomonas entomophila* in male *Drosophila melanogaster* . BMC Evolutionary Biology, 13, 185. 10.1186/1471-2148-13-185 24010544 PMC3847581

[ece37774-bib-0018] Gupta, V. , Venkatesan, S. , Chatterjee, M. , Syed, Z. A. , Nivsarkar, V. , & Prasad, N. G. (2016). No apparent cost of evolved immune response in *Drosophila melanogaster* . Evolution; International Journal of Organic Evolution, 70(4), 934–943. 10.1111/evo.12896 26932243

[ece37774-bib-0019] Hall, A. R. , Scanlan, P. D. , & Buckling, A. (2011). Bacteria‐phage coevolution and the emergence of generalist pathogens. The American Naturalist, 177(1), 44–53. 10.1086/657441 21117957

[ece37774-bib-0020] Kassen, R. (2002). The experimental evolution of specialists, generalists, and the maintenance of diversity. Journal of Evolutionary Biology, 15(2), 173–190. 10.1046/j.1420-9101.2002.00377.x

[ece37774-bib-0021] Kawecki, T. J. , Lenski, R. E. , Ebert, D. , Hollis, B. , Olivieri, I. , & Whitlock, M. C. (2012). Experimental evolution. Trends in Ecology & Evolution, 27(10), 547–560. 10.1016/j.tree.2012.06.001 22819306

[ece37774-bib-0022] Khalil, S. , Jacobson, E. , Chambers, M. C. , & Lazzaro, B. P. (2015). Systemic bacterial infection and immune defense phenotypes in *Drosophila melanogaster* . Journal of Visualized Experiments, 99, e52613. 10.3791/52613 PMC4542538 25992475

[ece37774-bib-0023] Khan, I. , Prakash, A. , & Agashe, D. (2017). Experimental evolution of insect immune memory versus pathogen resistance. Proceedings, 284, 20171583. 10.1098/rspb.2017.1583 PMC5745399 29237849

[ece37774-bib-0024] Kloesener, M. H. , Bose, J. , & Schulte, R. D. (2017). Experimental evolution with a multicellular host causes diversification within and between microbial parasite populations‐Differences in emerging phenotypes of two different parasite strains. Evolution; International Journal of Organic Evolution, 71(9), 2194–2205. 10.1111/evo.13306 28714591

[ece37774-bib-0025] Koskella, B. , Thompson, J. N. , Preston, G. M. , & Buckling, A. (2011). Local biotic environment shapes the spatial scale of bacteriophage adaptation to bacteria. The American Naturalist, 177(4), 440–451. 10.1086/658991 21460566

[ece37774-bib-0026] Kraaijeveld, A. R. , & Godfray, H. C. (1997). Trade‐off between parasitoid resistance and larval competitive ability in *Drosophila melanogaster* . Nature, 389(6648), 278–280. 10.1038/38483 9305840

[ece37774-bib-0027] Lazzaro, B. P. , Sceurman, B. K. , & Clark, A. G. (2004). Genetic basis of natural variation in *D. melanogaster* antibacterial immunity. Science (New York, N.Y.), 303(5665), 1873–1876. 10.1126/science.1092447 15031506

[ece37774-bib-0028] Leitão, A. B. , Arunkumar, R. , Day, J. P. , Geldman, E. M. , Morin‐Poulard, I. , Crozatier, M. , & Jiggins, F. M. (2020). Constitutive activation of cellular immunity underlies the evolution of resistance to infection in Drosophila. eLife, 9, 10.7554/eLife.59095 PMC7785293 33357377

[ece37774-bib-0029] Lively, C. M. , & Dybdahl, M. F. (2000). Parasite adaptation to locally common host genotypes. Nature, 405(6787), 679–681. 10.1038/35015069 10864323

[ece37774-bib-0030] Martinez, J. , Bruner‐Montero, G. , Arunkumar, R. , Smith, S. C. L. , Day, J. P. , Longdon, B. , & Jiggins, F. M. (2019). Virus evolution in *Wolbachia*‐infected *Drosophila* . Proceedings, 286, 20192117. 10.1098/rspb.2019.2117 PMC6823055 31662085

[ece37774-bib-0031] Masri, L. , Branca, A. , Sheppard, A. E. , Papkou, A. , Laehnemann, D. , Guenther, P. S. , Prahl, S. , Saebelfeld, M. , Hollensteiner, J. , Liesegang, H. , Brzuszkiewicz, E. , Daniel, R. , Michiels, N. K. , Schulte, R. D. , Kurtz, J. , Rosenstiel, P. , Telschow, A. , Bornberg‐Bauer, E. , & Schulenburg, H. (2015). Host‐pathogen coevolution: The selective advantage of *Bacillus thuringiensis* virulence and its cry toxin genes. PLoS Biology, 13(6), e1002169. 10.1371/journal.pbio.1002169 26042786 PMC4456383

[ece37774-bib-0032] Morran, L. T. , Parrish, R. C. , Gelarden, I. A. , Allen, M. B. , & Lively, C. M. (2014). Experimental coevolution: Rapid local adaptation by parasites depends on host mating system. The American Naturalist, 184(Suppl 1), S91–100. 10.1086/676930 PMC4180910 25061681

[ece37774-bib-0033] Morran, L. T. , Schmidt, O. G. , Gelarden, I. A. , Parrish, R. C. , & Lively, C. M. (2011). Running with the Red Queen: Host‐parasite coevolution selects for biparental sex. Science (New York, N.Y.), 333(6039), 216–218. 10.1126/science.1206360 PMC3402160 21737739

[ece37774-bib-0034] Mukherjee, K. , Grizanova, E. , Chertkova, E. , Lehmann, R. , Dubovskiy, I. , & Vilcinskas, A. (2017). Experimental evolution of resistance against *Bacillus thuringiensis* in the insect model host *Galleria mellonella* results in epigenetic modifications. Virulence, 8(8), 1618–1630. 10.1080/21505594.2017.1325975 28521626 PMC5810480

[ece37774-bib-0035] Poullain, V. , Gandon, S. , Brockhurst, M. A. , Buckling, A. , & Hochberg, M. E. (2008). The evolution of specificity in evolving and coevolving antagonistic interactions between a bacteria and its phage. Evolution; International Journal of Organic Evolution, 62(1), 1–11. 10.1111/j.1558-5646.2007.00260.x 18005153

[ece37774-bib-0036] Rafaluk, C. , Yang, W. , Mitschke, A. , Rosenstiel, P. , Schulenburg, H. , & Joop, G. (2017). Highly potent host external immunity acts as a strong selective force enhancing rapid parasite virulence evolution. Environmental Microbiology, 19(5), 2090–2100. 10.1111/1462-2920.13736 28345225

[ece37774-bib-0037] Rafaluk‐Mohr, C. , Wagner, S. , & Joop, G. (2018). Cryptic changes in immune response and fitness in *Tribolium castaneum* as a consequence of coevolution with *Beauveria bassiana* . Journal of Invertebrate Pathology, 152, 1–7. 10.1016/j.jip.2017.12.003 29273219

[ece37774-bib-0038] Remold, S. (2012). Understanding specialism when the Jack of all trades can be the master of all. Proceedings. Biological Sciences, 279(1749), 4861–4869. 10.1098/rspb.2012.1990 23097515 PMC3497242

[ece37774-bib-0039] Rose, M. R. (1984). Laboratory evolution of postponed senescence in *Drosophila melanogaster* . Evolution; International Journal of Organic Evolution, 38(5), 1004–1010. 10.1111/j.1558-5646.1984.tb00370.x 28555803

[ece37774-bib-0040] Therneau, T. M. (2020). coxme: Mixed Effects Cox Models. Available at: https://CRAN.R‐project.org/package=coxme Accessed: 16 January 2021

[ece37774-bib-0041] Tinsley, M. C. , Blanford, S. , & Jiggins, F. M. (2006). Genetic variation in *Drosophila melanogaster* pathogen susceptibility. Parasitology, 132(Pt 6), 767–773. 10.1017/S0031182006009929 16497252 PMC1847563

[ece37774-bib-0042] Woolhouse, M. E. J. , Webster, J. P. , Domingo, E. , Charlesworth, B. , & Levin, B. R. (2002). Biological and biomedical implications of the co‐evolution of pathogens and their hosts. Nature Genetics, 32(4), 569–577. 10.1038/ng1202-569 12457190

[ece37774-bib-0043] Zaman, L. , Meyer, J. R. , Devangam, S. , Bryson, D. M. , Lenski, R. E. , & Ofria, C. (2014). Coevolution drives the emergence of complex traits and promotes evolvability. PLoS Biology, 12(12), e1002023. 10.1371/journal.pbio.1002023 25514332 PMC4267771

