# Supplementary material for: Enemies make you stronger: Coevolution between fruit fly host and bacterial pathogen increases postinfection survivorship in the host
Source: Ecol Evol. 2021 Jun 22;11(14):9563–74. doi: 10.1002/ece3.7774 (PMC8293768; doi:10.1002/ece3.7774)
Supplement: Supplementary file 1 — Supplementary Material [file ECE3-11-9563-s001.docx]

**Figure S1.** Survivorship curves (plotted separately for each block) for male (a) and female (b) hosts from Adapt (red), Co.S (purple) or Coev (blue) regimes infected with either Anc Pe (solid curves) or the Coev Pe (dotted curves) from the corresponding block after 20 cycles of coevolution (experiment 1).

(a)

****(b)

**Figure S2.** Survivorship curves for female (panel on the left) and male (panel on the right) hosts from Adapt (red), Co.S (purple), Co.U (black) or Coev (blue) regimes infected with either Anc Pe (solid curves) or the Coev Pe (dotted curves) from the corresponding block after 20 cycles of coevolution (experiment 1).

**Figure S3.** Survivorship curves (plotted separately for each block) for male hosts (a) and female host (b) from Adapt (red), Co.S (purple), Co.U (black) or Coev (blue) regimes infected with either Anc Pe (solid curves) or the Coev Pe (dotted curves) from the corresponding block after 20 cycles of coevolution (experiment 1).

(a)

(b)

**Figure S4.** Mean proportion of flies surviving 96 hours post infection with Ancestral Pe after 10, 15 and 20 cycles of coevolution. Adapt (red), Co.S (purple), Co.U (black) and Coev (blue). ****

**Table S1.** The output of Cox’s proportional hazards models for male and female hosts from Adapt, Coev and Co.S populations from block 1 infected with either Anc Pe or the block 1 Coev Pe (experiment 1). Hazard rates are expressed relative to the hazard rates of the default level of each fixed factor, which are constrained to be 1. The default level for “Selection” is Adapt, while the default level for “Pathogen” is Anc Pe. Lower CI and Upper CI indicate lower and upper bounds of 95% confidence intervals. Confidence intervals that do not contain 1 signify statistical significance and are shown in bold. Higher hazard rates are equivalent to lower survivorship in the hosts.

| **A) Block 1 Females** | | | |
| --- | --- | --- | --- |
| **Fixed Coefficients** | **Hazard Rates** | **Lower CI** | **Upper CI** |
| Selection Co.S | 2.9571 | **1.5715** | **5.5643** |
| Selection Coev | 0.2551 | **0.0840** | **0.7752** |
| Pathogen Coev Pe | 0.9613 | 0.4518 | 2.0453 |
| Selection Co.S : Pathogen Coev Pe | 3.0739 | **1.2707** | **7.4362** |
| Selection Coev : Pathogen Coev Pe | 2.2166 | 0.5369 | 9.1518 |
|  |  |  |  |
| **B) Block 1 Males** | | | |
| **Fixed Coefficients** | **Hazard Rates** | **Lower CI** | **Upper CI** |
| Selection Co.S | 6.2017 | **2.5652** | **14.9930** |
| Selection Coev | 0.3204 | 0.0647 | 1.5870 |
| Pathogen Coev Pe | 2.2196 | 0.8330 | 5.9140 |
| Selection Co.S : Pathogen Coev Pe | 1.0449 | 0.3505 | 3.1150 |
| Selection Coev : Pathogen Coev Pe | 0.6769 | 0.0880 | 5.2060 |

**Table S2.** The output of Cox’s proportional hazards models for male and female hosts from Adapt., Coev and Co.S populations from block 2 infected with either Anc Pe or the block 2 Coev Pe (experiment 1). Hazard rates are expressed relative to the hazard rates of the default level of each fixed factor, which are constrained to be 1. The default level for “Selection” is Adapt., while the default level for “Pathogen” is Anc Pe. Lower CI and Upper CI indicate lower and upper bounds of 95% confidence intervals. Confidence intervals that do not contain 1 signify statistical significance and are shown in bold. Higher hazard rates are equivalent to lower survivorship in the hosts.

| **A) Block 2 Females** | | | |
| --- | --- | --- | --- |
| **Fixed Coefficients** | **Hazard Rates** | **Lower CI** | **Upper CI** |
| Selection Co.S | 10.0947 | **3.5464** | **28.7346** |
| Selection Coev | 0.7358 | 0.1647 | 3.2876 |
| Pathogen Coev Pe | 18.8604 | **6.7480** | **52.7146** |
| Selection Co.S : Pathogen Coev Pe | 0.2135 | **0.0689** | **0.6617** |
| Selection Coev : Pathogen Coev Pe | 0.4546 | 0.0931 | 2.2209 |
|  |  |  |  |
| **B) Block 2 Males** | | | |
| **Fixed Coefficients** | **Hazard Rates** | **Lower CI** | **Upper CI** |
| Selection Co.S | 3.4533 | **1.5370** | **7.7590** |
| Selection Coev | 0.3522 | 0.0934 | 1.3280 |
| Pathogen Coev Pe | 4.4839 | **2.0418** | **9.8470** |
| Selection Co.S : Pathogen Coev Pe | 1.1381 | 0.4436 | 2.9200 |
| Selection Coev : Pathogen Coev Pe | 1.2403 | 0.2840 | 5.4160 |

**Table S3.** The output of Cox’s proportional hazards models for male and female hosts from Adapt., Coev and Co.S populations from block 3 infected with either Anc Pe or the block 3 Coev Pe (experiment 1). Hazard rates are expressed relative to the hazard rates of the default level of each fixed factor, which are constrained to be 1. The default level for “Selection” is Adapt., while the default level for “Pathogen” is Anc Pe. Lower CI and Upper CI indicate lower and upper bounds of 95% confidence intervals. Confidence intervals that do not contain 1 signify statistical significance and are shown in bold. Higher hazard rates are equivalent to lower survivorship in the hosts.

| **A) Block 3 Females** | | | |
| --- | --- | --- | --- |
| **Fixed Coefficients** | **Hazard Rates** | **Lower CI** | **Upper CI** |
| Selection Co.S | 10.9632 | 3.3081 | 36.3330 |
| Selection Coev | 2.8715 | 0.7618 | 10.8240 |
| Pathogen Coev Pe | 2.7324 | 0.7249 | 10.3000 |
| Selection Co.S : Pathogen Coev Pe | 0.9843 | 0.2386 | 4.0610 |
| Selection Coev : Pathogen Coev Pe | 0.3062 | 0.0576 | 1.6270 |
|  |  |  |  |
| **B) Block 3 Males** | | | |
| **Fixed Coefficients** | **Hazard Rates** | **Lower CI** | **Upper CI** |
| Selection Co.S | 1.4973 | 0.7336 | 3.0560 |
| Selection Coev | 0.3483 | 0.1242 | 0.9771 |
| Pathogen Coev Pe | 0.8674 | 0.3958 | 1.9010 |
| Selection Co.S : Pathogen Coev Pe | 7.7858 | 2.9734 | 20.3869 |
| Selection Coev : Pathogen Coev Pe | 1.1177 | 0.2577 | 4.8468 |

**Table S4.** The output of Cox’s proportional hazards models for male and female hosts from Adapt., Coev and Co.S populations from block 4 infected with either Anc Pe or the block 4 Coev Pe (experiment 1). Hazard rates are expressed relative to the hazard rates of the default level of each fixed factor, which are constrained to be 1. The default level for “Selection” is Adapt., while the default level for “Pathogen” is Anc Pe. Lower CI and Upper CI indicate lower and upper bounds of 95% confidence intervals. Confidence intervals that do not contain 1 signify statistical significance and are shown in bold. Higher hazard rates are equivalent to lower survivorship in the hosts.

| **A) Block 4 Females** | | | |
| --- | --- | --- | --- |
| **Fixed Coefficients** | **Hazard Rates** | **Lower CI** | **Upper CI** |
| Selection Co.S | 1.8981 | 1.0530 | 3.4210 |
| Selection Coev | 0.5074 | 0.2342 | 1.0990 |
| Pathogen Coev Pe | 1.0672 | 0.5601 | 2.0340 |
| Selection Co.S : Pathogen Coev Pe | 0.6018 | 0.2570 | 1.4090 |
| Selection Coev : Pathogen Coev Pe | 0.6923 | 0.2233 | 2.1460 |
|  |  |  |  |
| **B) Block 4 Males** | | | |
| **Fixed Coefficients** | **Hazard Rates** | **Lower CI** | **Upper CI** |
| Selection Co.S | 1.1410 | 0.6843 | 1.9025 |
| Selection Coev | 0.1195 | 0.0461 | 0.3097 |
| Pathogen Coev Pe | 0.6631 | 0.3765 | 1.1677 |
| Selection Co.S : Pathogen Coev Pe | 1.1733 | 0.5473 | 2.5153 |
| Selection Coev : Pathogen Coev Pe | 0.9025 | 0.1936 | 4.2061 |

**Table S5.** The output of Cox’s proportional hazards models for the flies from Adapt, Coev and Co.S and Co.U selection regimes from each block infected with either Anc Pe or the Coev Pe (experiment 1). Hazard rates are expressed relative to the hazard rates of the default level of each factor which are constrained to be 1. The default level for “Selection” is Adapt, while the default level for “Pathogen” is Anc Pe. Lower CI and Upper CI indicate lower and upper bounds of 95% confidence intervals. Confidence intervals that do not contain 1 signify statistical significance and are shown in bold. Higher hazard rates are equivalent to lower survivorship in the hosts.

| **A) FEMALES** |  |  |  |
| --- | --- | --- | --- |
|  |  |  |  |
| **Fixed Coefficients** | **Hazard Ratios** | **Lower CI** | **Upper CI** |
| SelectionCo.S | 3.8229283 | **2.6571** | **5.5011** |
| SelectionCo.U | 5.5410692 | **3.8811** | **7.9111** |
| SelectionCoev | 0.6193364 | 0.3751 | 1.0231 |
| PathogenCoev Pe | 2.6570754 | **1.8151** | **3.8911** |
| SelectionCo.S:PathogenCoev Pe | 0.8618343 | 0.5491 | 1.3541 |
| SelectionCo.U:PathogenCoev Pe | 0.8487869 | 0.5441 | 1.3231 |
| SelectionCoev:PathogenCoev Pe | 0.7175561 | 0.3861 | 1.3361 |
|  |  |  |  |
| **Randon Effects** | **Variance** |  |  |
| Block | 0.1789 |  |  |
|  |  |  |  |
| **B) MALES** |  |  |  |
| **Fixed Coefficients** | **Hazard Ratios** | **Lower CI** | **Upper CI** |
| SelectionCo.S | 2.1171 | **1.5221** | **2.9441** |
| SelectionCo.U | 3.6341 | **2.6551** | **4.9741** |
| SelectionCoev | 0.2441 | **0.1381** | **0.4321** |
| PathogenCoev Pe | 1.4301 | **1.0071** | **2.0301** |
| SelectionCo.S:PathogenCoev Pe | 1.8921 | **1.2301** | **2.9121** |
| SelectionCo.U:PathogenCoev Pe | 1.7311 | **1.1401** | **2.6271** |
| SelectionCoev:PathogenCoev Pe | 1.2221 | 0.5891 | 2.5341 |
|  |  |  |  |
| **Randon Effects** | **Variance** |  |  |
| Block | 0.0812 |  |  |

**Table S6.** The output of Cox’s proportional hazards models for the flies from Adapt, Coev and Co.S and Co.U selection regimes from each block infected with either Anc Pe or the Coev Pe (experiment 1). Hazard rates are expressed relative to the hazard rates of the default level of each fixed factor, which are constrained to be 1. The default level for “Selection” is Adapt, the default level for “Pathogen” is Anc Pe, the default level for “Sex” is Females “F” while the default level for “Block” is Block 1 “1”. Lower CI and Upper CI indicate lower and upper bounds of 95% confidence intervals. Confidence intervals that do not contain 1 signify statistical significance and are shown in bold. Higher hazard rates are equivalent to lower survivorship in the hosts.

| **Fixed Coefficients** | **Hazard ratio** | **Lower CI** | **Upper CI** |
| --- | --- | --- | --- |
| Selection Co.S | 2.7693 | 1.473166 | 5.20611 |
| Selection Co.U | 3.4893 | 1.877114 | 6.486354 |
| Selection Coev | 0.2604 | 0.085727 | 0.791219 |
| Pathogen Coev Pe | 0.9580 | 0.450351 | 2.03827 |
| Sex M | 0.4052 | 0.155729 | 1.054543 |
| Block 2 | 0.2528 | 0.083219 | 0.768325 |
| Block 3 | 0.1964 | 0.056441 | 0.683419 |
| Block 4 | 1.3361 | 0.664434 | 2.686948 |
| Selection Co.S:Pathogen Coev Pe | 2.7243 | 1.130117 | 6.567568 |
| Selection Co.U:Pathogen Coev Pe | 2.7512 | 1.149365 | 6.585485 |
| Selection Coev:Pathogen Coev Pe | 2.1955 | 0.531802 | 9.064133 |
| Selection Co.S:Sex M | 2.3127 | 0.782009 | 6.839772 |
| Selection Co.U:Sex M | 2.5679 | 0.886544 | 7.438439 |
| Selection Coev:Sex M | 1.2256 | 0.174679 | 8.59965 |
| Pathogen Coev Pe:Sex M | 2.3306 | 0.676463 | 8.030132 |
| Selection Co.S:Block 2 | 3.6038 | 1.062764 | 12.22099 |
| Selection Co.U:Block 2 | 4.2650 | 1.281524 | 14.19426 |
| Selection Coev:Block 2 | 2.8264 | 0.438108 | 18.23446 |
| Selection Co.S:Block 3 | 3.7650 | 0.972446 | 14.57686 |
| Selection Co.U:Block 3 | 5.1858 | 1.361447 | 19.75291 |
| Selection Coev:Block 3 | 10.9415 | 1.93833 | 61.76304 |
| Selection Co.S:Block 4 | 0.6494 | 0.274089 | 1.538891 |
| Selection Co.U:Block 4 | 0.7318 | 0.315 | 1.700184 |
| Selection Coev:Block 4 | 1.9630 | 0.507064 | 7.600011 |
| Pathogen Coev Pe:Block 2 | 19.2729 | 5.396523 | 68.83104 |
| Pathogen Coev Pe:Block 3 | 2.8212 | 0.612977 | 12.98531 |
| Pathogen Coev Pe:Block 4 | 1.0964 | 0.406308 | 2.958917 |
| Sex M:Block 2 | 5.2335 | 1.127969 | 24.28239 |
| Sex M:Block 3 | 12.0923 | 2.495262 | 58.60141 |
| Sex M:Block 4 | 5.0735 | 1.647378 | 15.62535 |
| Selection Co.S:Pathogen Coev Pe:Sex M | 0.4013 | 0.098795 | 1.630758 |
| Selection Co.U:Pathogen Coev Pe:Sex M | 0.5267 | 0.132133 | 2.099654 |
| Selection Coev:Pathogen Coev Pe:Sex M | 0.3066 | 0.025565 | 3.677318 |
| Selection Co.S:Pathogen Coev Pe:Block 2 | 0.0774 | 0.018521 | 0.323818 |
| Selection Co.U:Pathogen Coev Pe:Block 2 | 0.1164 | 0.028398 | 0.477733 |
| Selection Coev:Pathogen Coev Pe:Block 2 | 0.2096 | 0.024984 | 1.758381 |
| Selection Co.S:Pathogen Coev Pe:Block 3 | 0.3407 | 0.064314 | 1.80493 |
| Selection Co.U:Pathogen Coev Pe:Block 3 | 0.1803 | 0.034408 | 0.945486 |
| Selection Coev:Pathogen Coev Pe:Block 3 | 0.1421 | 0.01589 | 1.270943 |
| Selection Co.S:Pathogen Coev Pe:Block 4 | 0.2353 | 0.06921 | 0.799941 |
| Selection Co.U:Pathogen Coev Pe:Block 4 | 0.2826 | 0.086175 | 0.926915 |
| Selection Coev:Pathogen Coev Pe:Block 4 | 0.3264 | 0.053214 | 2.002672 |
| Selection Co.S:Sex M:Block 2 | 0.1586 | 0.028705 | 0.877156 |
| Selection Co.U:Sex M:Block 2 | 0.1350 | 0.025219 | 0.722973 |
| Selection Coev:Sex M:Block 2 | 03856 | 0.023632 | 6.293665 |
| Selection Co.S:Sex M:Block 3 | 0.0615 | 0.010523 | 0.35984 |
| Selection Co.U:Sex M:Block 3 | 0.1594 | 0.02887 | 0.880608 |
| Selection Coev:Sex M:Block 3 | 0.1006 | 0.00768 | 1.318872 |
| Selection Co.S:Sex M:Block 4 | 0.2828 | 0.074411 | 1.074951 |
| Selection Co.U:Sex M:Block 4 | 0.1678 | 0.045252 | 0.62283 |
| Selection Coev:Sex M:Block 4 | 0.1830 | 0.018319 | 1.829498 |
| Pathogen Coev Pe:Sex M:Block 2 | 0.1166 | 0.019512 | 0.697434 |
| Pathogen Coev Pe:Sex M:Block 3 | 0.1369 | 0.018968 | 0.988065 |
| Pathogen Coev Pe:Sex M:Block 4 | 0.2706 | 0.060077 | 1.219624 |
| Selection Co.S:Pathogen Coev Pe:Sex M:Block 2 | 14.1669 | 1.863866 | 107.6809 |
| Selection Co.U:Pathogen Coev Pe:Sex M:Block 2 | 10.8839 | 1.481232 | 79.9739 |
| Selection Coev:Pathogen Coev Pe:Sex M:Block 2 | 8.1708 | 0.302851 | 220.4457 |
| Selection Co.S:Pathogen Coev Pe:Sex M:Block 3 | 19.2769 | 2.117076 | 175.5261 |
| Selection Co.U:Pathogen Coev Pe:Sex M:Block 3 | 11.1800 | 1.285704 | 97.21829 |
| Selection Coev:Pathogen Coev Pe:Sex M:Block 3 | 11.7485 | 0.418941 | 329.4675 |
| Selection Co.S:Pathogen Coev Pe:Sex M:Block 4 | 4.4959 | 0.737125 | 27.42229 |
| Selection Co.U:Pathogen Coev Pe:Sex M:Block 4 | 3.3407 | 0.567525 | 19.66486 |
| Selection Coev:Pathogen Coev Pe:Sex M:Block 4 | 4.0997 | 0.178544 | 94.13718 |
|  |  |  |  |

**Table S7.** Analysis of Variance table showing the effects of Selection (Coev, Adapt, Co.S and Co.U), Generation and their interaction in males and females from the selection experiment conducted after generation 10^th^, 15^th^ and 20^th^.

| **Type III Analysis of Variance Table with Satterthwaite's method** | | | | | | |
| --- | --- | --- | --- | --- | --- | --- |
| **A) Females** |  |  |  |  |  |  |
| **Effect** | **SS** | **MS** | **NumDF** | **DenDF** | **F value** | **p value** |
| Selection | 1.77377 | 0.59126 | 3 | 33 | 42.9831 | **0.0001** |
| Generation | 0.20052 | 0.10026 | 2 | 33 | 7.2891 | **0.0021** |
| Selection:Generation | 0.23765 | 0.03961 | 6 | 33 | 2.8791 | **0.0231** |
|  |  |  |  |  |  |  |
| **Type III Analysis of Variance Table with Satterthwaite's method** | | | | |  |  |
| **B) Males** |  |  |  |  |  |  |
| **Effect** | **SS** | **MS** | **NumDF** | **DenDF** | **F value** | **p value** |
| Selection | 1.81357 | 0.60452 | 3 | 32.304 | 18.1411 | **0.0001** |
| Generation | 0.10267 | 0.05134 | 2 | 34.821 | 1.5411 | 0.2291 |
| Selection:Generation | 0.16726 | 0.02788 | 6 | 32.304 | 0.8371 | 0.5511 |
